# Unusual source of tachycardia in an adolescent

**DOI:** 10.1186/1865-1380-4-9

**Published:** 2011-03-16

**Authors:** Marvin B Mata, Brian T Kloss, Jennifer A Campoli, Karen Teelin

**Affiliations:** 1 Department of Pediatrics, SUNY Upstate Medical University, Syracuse, NY, USA; 2 Department of Emergency Medicine, SUNY Upstate Medical University, Syracuse, NY, USA

## Abstract

Mahaim fiber tachycardia is an uncommon cause of palpitations among the pediatric population. This case report describes an adolescent female who presented with recurrent episodes of tachycardia with chest pain and dizziness. Her ECG showed tachycardia with wide QRS complexes and left bundle branch block pattern. Repeat ECG after adenosine treatment revealed sinus rhythm with persistence of the left bundle branch block pattern. Metoprolol was started however she continued to have episodes of sustained tachycardia.

Electrophysiologic study then confirmed the diagnosis of Mahaim fiber tachycardia. Treatment was successful with mapping of the accessory pathways followed by radiofrequency ablation.

## Introduction

Patients who present with wide complex tachycardia are always challenging both diagnostically and therapeutically. There can be disagreement among physicians over the ECG interpretation and the best treatment option for the patient. Mahaim fiber is an uncommon cause of tachycardia in which cardiac pre-excitation occurs via slow-conducting, long accessory pathways that terminate in the right ventricular free wall or into the adjacent right bundle. It was first reported by Mahaim and Bennett who found accessory conducting tissues that originated from the Bundle of His and terminated in the right ventricle. Subsequently, other investigators have elucidated the electrophysiologic properties of this pathway leading to the currently accepted concept of slow and decremental anterograde fiber conduction.

## Case report

A 17-year-old previously healthy female presented to the emergency department in the early morning hours with a feeling that her heart was racing. Her symptoms had been ongoing for several hours with accompanying shortness of breath, lightheadedness, nausea, and vomiting. She had experienced recurrent palpitations that usually spontaneously resolved within 20 min over the past year. On the evening prior to presentation, she had drunk multiple cans of caffeinated soda and was up most of the night. She denied drug and alcohol use, fever, recent illness, or any other significant past medical history.

**Figure 1:**
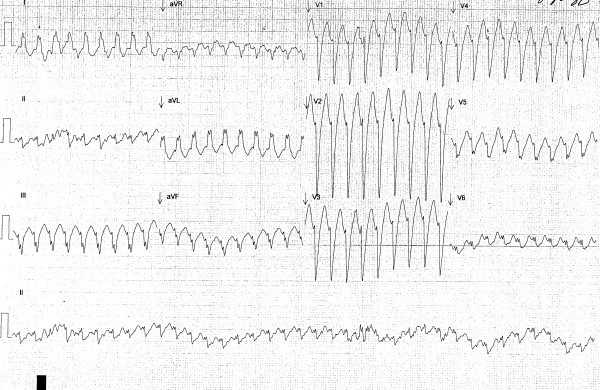
**The electrocardiogram (*ECG*) revealed a wide complex tachycardia with left bundle branch morphology, a superior axis, an rS in lead III, an R wave in V1, and late QRS transition (after V5)**.

**Figure 2:**
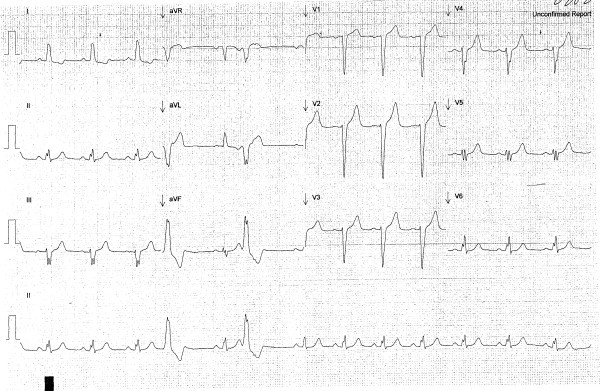
**The repeat ECG showed a sinus rhythm with premature ventricular complexes and left bundle branch block morphology**.

At presentation, her heart rate was 220 beats per minute. The electrocardiogram (ECG) revealed a wide complex tachycardia with left bundle branch morphology, a superior axis, an rS in lead III, an R wave in V1, and late QRS transition (after V5) (Figure 1). She was treated with 6 mg rapid-infusion adenosine intravenously, which reduced her heart rate to 78 beats per minute. The repeat ECG showed a sinus rhythm with premature ventricular complexes and left bundle branch block morphology (Figure 2).

Urine toxicology screen, complete blood count, complete metabolic panel, thyroid function tests, and beta-HCG laboratory work all came back within normal limits.

The patient was hospitalized for additional monitoring and remained stable on Metoprolol. Just prior to discharge she developed another episode of sustained tachycardia of the same character, which reverted back to sinus rhythm after the administration of adenosine. She was then discharged on extended release Metoprolol, 25 mg PO daily. A cardiac catheterization with an electrophysiologic study was scheduled to be performed on an outpatient basis.

Electrophysiology showed normal baseline conduction intervals. However, on rapid atrial pacing she demonstrated the same widened QRS complexes with left bundle branch pattern and axis of about 0 degrees on the frontal plane without PR shortening. Careful mapping showed triple Mahaim potentials along the right lateral tricuspid annulus with slow conduction of the right accessory pathways, which resolved with the administration of adenosine. These findings were highly suggestive of Mahaim fiber accessory pathway and were successfully treated with radiofrequency ablation.

## Discussion

The patient described in this report is a previously healthy adolescent who presented with recurrent episodes of tachycardia felt as palpitations with or without chest pain and lightheadedness. Clinical presentation of Mahaim fiber tachycardia varies widely and ranges from the asymptomatic to symptomatic arrhythmias with palpitations, light headedness, chest pain, syncope, and even sudden cardiac death. Associated conditions may include Ebstein's anomaly, atrial septal defect, hypertrophic cardiomyopathy, rheumatic heart disease, Klippel-Feil syndrome, anomalous origin of the left main coronary artery to the right aortic sinus, partial anomalous pulmonary venous return, Rett syndrome, and coronary artery disease. Although there are no clinical variables that highly correlate SVT with aberrancy, a focused history and physical examination help to rule out other etiologies. Tachycardia coupled with the presence of cannon A waves due to AV dissociation point to a ventricular rather than a supraventricular cause. As in this case, an adolescent without any significant prior medical history is more likely to have supraventricular tachycardia; however, neither age nor gender is sensitive or specific enough to determine the underlying etiology of a wide complex tachycardia alone.

Normal heart conduction occurs from the sino-atrial (SA) node in the atrium to the atrioventricular (AV) node, and then progresses along the His Purkinje fibers of the ventricle. Anatomically, Mahaim fibers originate from the right atrium along the tricuspid annulus and insert distally into the right ventricle free wall or near the right bundle branch [1,2]. Ventricular endocardial mapping has identified the more common forms as atriofascicular and atrioventicular tracts [3].

When the patient was in sinus rhythm, the ECG revealed an rS pattern in lead III and persistent left bundle branch block at slower rates, whereas a wide complex QRS with left bundle branch block pattern and superior axis was observed during tachycardia. The differential diagnoses for wide complex tachycardia include SVT with aberrancy, ventricular tachycardia, Wolff Parkinson White Syndrome, electrolyte abnormality, and drug toxicity, among others. However, there was no AV dissociation noted, and the left bundle branch block pattern provided a significant clue leading to the correct diagnosis.

Normal atrioventicular conduction usually occurs at a slow rate, and so ECG findings in sinus rhythm may be normal. The accessory pathways are not activated because of their slower rate. Subtle but very important clues to the diagnosis of Mahaim fiber include the absence of Q wave in leads V5 or V6, or a narrow QRS with an rS pattern in lead III during sinus rhythm with a left axis deviation [3,4]. Other reported findings for the atriofascicular pathway include the QRS axis between 0 and -75°, QRS width ≤ 0.15 s, an R wave in lead I, an rS pattern in lead V_1_, RS > 1 QRS transition > V_4_, and cycle length between 220 and 450 ms with 87.5% sensitivity [5].

With rapid heart rates conduction occurs through the accessory pathway. Most of the conduction then happens along the accessory pathways, which depolarizes the right ventricular myocardium first then spreads to the left. The patient's electrophysiology study later showed an antidromic atrioventricular reentry tachycardia (AVRT) with antegrade conduction through the accessory pathway and retrograde conduction via the AV nodal axis. All of these explain the more common finding of left bundle branch block morphology with widened QRS complex during tachycardia due to Mahaim fiber activation as observed in this patient. In addition, dual AV nodal pathways were noted. This finding is present in as many as 85% of patients with this disorder [6].

Pharmacologic response to quinidine, digoxin, propranolol, and adenosine has been reported. The patient's response to adenosine and the development of ventricular extrasystoles after the treatment suggest possible AV nodal properties of the Mahaim fiber with possible spontaneous automaticity. Adenosine is a purine nucleoside that acts on the adenosine 1 receptor causing a conduction blockade at the AV node. The administration of this drug necessitates cardiac monitoring as adenosine can shorten the refractory period of accessory pathways and in Mahaim fiber tachycardia can potentially precipitate atrial fibrillation.

Radiofrequency ablation remains the treatment of choice for this disorder. Mapping the accessory fibers identifies the proximal and distal insertion of the fibers to allow for successful ablation [1,7-9].

## Conclusion

Mahaim fiber tachycardia is an uncommon cause of tachycardia in children. ECG shows tachycardia with widened QRS complexes and left bundle branch block pattern, which in this case responded to rapid intravenous adenosine infusion. Electrophysiologic mapping of the fibers is the key to diagnosis and successful ablation.

This case report met the criteria for an exemption for review by the Institutional Review Board for the Protection of Human Subjects at SUNY Upstate Medical University.

## Consent

This case report qualifies as for an IRB exemption from the SUNY Upstate Medical University IRB Board given its nature as a case report wherein no patient identifiers are disclosed or revealed in the publication process. A copy of the IRB exemption policy is available for review by the Editor-in-Chief of this journal. Signed consent from the patient was obtained.

## Competing interests

The authors declare that they have no competing interests.

## Authors' contributions

MM served as the first author. BK oversaw the collection and editing of the ECG images as well as served as corresponding author. KT proof read the paper and JC oversaw the organization of the paper.
